# Novel enzyme‐resistant pancreatic polypeptide analogs evoke pancreatic beta‐cell rest, enhance islet cell turnover, and inhibit food intake in mice

**DOI:** 10.1002/biof.2059

**Published:** 2024-04-18

**Authors:** Wuyun Zhu, Neil Tanday, Ryan A. Lafferty, Peter R. Flatt, Nigel Irwin

**Affiliations:** ^1^ Diabetes Research Centre Schools of Biomedical Sciences and Pharmacy & Pharmaceutical Sciences, Ulster University Coleraine UK

**Keywords:** apoptosis, beta‐cell, pancreatic polypeptide (PP), proliferation, satiety

## Abstract

Pancreatic polypeptide (PP) is a postprandial hormone secreted from pancreatic islets that activates neuropeptide Y4 receptors (NPY4Rs). PP is known to induce satiety but effects at the level of the endocrine pancreas are less well characterized. In addition, rapid metabolism of PP by dipeptidyl peptidase‐4 (DPP‐4) limits the investigation of the effects of the native peptide. Therefore, in the present study, five novel amino acid substituted and/or fatty acid derivatized PP analogs were synthesized, namely [P^3^]PP, [K^13^Pal]PP, [P^3^,K^13^Pal]PP, [N‐Pal]PP, and [N‐Pal,P^3^]PP, and their impact on pancreatic beta‐cell function, as well as appetite regulation and glucose homeostasis investigated. All PP analogs displayed increased resistance to DPP‐4 degradation. In addition, all peptides inhibited alanine‐induced insulin secretion from BRIN‐BD11 beta cells. Native PP and related analogs (10^−8^ and 10^−6^ M), and especially [P^3^]PP and [K^13^Pal]PP, significantly protected against cytokine‐induced beta‐cell apoptosis and promoted cellular proliferation, with effects dependent on the NPY4R for all peptides barring [N‐Pal,P^3^]PP. In mice, all peptides, except [N‐Pal]PP and [N‐Pal,P^3^]PP, evoked a dose‐dependent (25, 75, and 200 nmol/kg) suppression of appetite, with native PP and [P^3^]PP further augmenting glucagon‐like peptide‐1 (GLP‐1) and cholecystokinin (CCK) induced reductions of food intake. The PP peptides had no obvious detrimental effect on glucose tolerance and they did not noticeably impair the glucose‐regulatory actions of GLP‐1 or CCK. In conclusion, Pro^3^ amino acid substitution of PP, either alone or together with mid‐chain acylation, creates PP analogs with benefits on beta‐cell rest, islet cell turnover, and energy regulation that may be applicable to the treatment of diabetes and obesity.

## Graphical abstract

Pancreatic polypeptide (PP) is a hormone secreted from pancreatic islets that have not been fully characterized. In the current study, we show the benefits of PP on pancreatic beta‐cell health as well as the induction of appetite suppression. Moreover, we have also created long‐acting PP analogs that retain the bioactivity profile of native PP, but with a more prolonged duration of action.

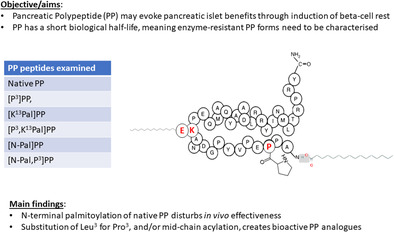

**Abbreviations**
CCK: cholecystokinin
DPP‐4: dipeptidyl peptidase‐4
GLP‐1: glucagon‐like peptide‐1
MALDI‐ToF MS: matrix‐assisted laser desorption ionization time‐of‐flight mass spectrometry
NPY: neuropeptide Y
NPY1R: neuropeptide Y1 receptor
NPY4R: neuropeptide Y4 receptor
PP: Pancreatic polypeptide
rp‐HPLC: reversed‐phased high‐performance liquid chromatography

## 1 INTRODUCTION

Pancreatic polypeptide (PP), a hormone of the neuropeptide Y (NPY) family, is released postprandially from pancreatic islet γ‐cells^1^ and exerts well‐documented effects on the regulation of energy balance.^2^ These biological actions are believed to be mediated through selective binding and activation of the neuropeptide Y4 receptor (NPY4R) that is expressed both centrally and peripherally.^3^ As a mediator of negative energy balance, a putative therapeutic role for PP in obesity was first suggested in 2003 following observations of appetite suppression in humans^4^ as well as the ability to elevate energy expenditure and induce weight loss.^2^ In agreement, elevated levels of PP are observed in patients with anorexia nervosa, with lower levels in Prader‐Willi syndrome where increased appetite is characteristic.^5^ More recently, human obesity and diabetes were shown to be associated with impaired postprandial PP secretion,^6^ suggesting the direct involvement of PP in the metabolic dysregulation of obesity. Furthermore, PP is known to delay gastric emptying,^7^ enhance insulin sensitivity^8^ and reverse hepatic insulin resistance,^9^ which together point toward clear benefits in obesity‐driven forms of diabetes.

To add to this, NPY4R activation has also been shown to exert positive effects on pancreatic beta‐cell survival.^10^ A similar pancreatic islet benefit has been described following activation of related neuropeptide Y1 receptors (NPY1R), that is linked to both growth of beta cells and protection against apoptosis^11^ as well as promotion of alpha‐ to beta‐cell transdifferentiation.^12^ Despite this, prolonged activation of NPY1R could stimulate appetite and induce hypertension,^13^, ^14^ with obvious problems for the treatment of obesity‐diabetes. In that regard, the NPY1R and NPY4R subtypes share notable sequence homology and the cell signaling pathways activated are equivalent, being linked to G_iα_ stimulation that lowers cAMP and the activity of its downstream targets, PKA and Epac2.^15^ Thus, comparable benefits on beta‐cell turnover for NPY1R and NPY4R activation would be expected, with further advantages of NPY4R over NPY1R to induce satiety rather than hunger^4^ as well as lacking an impact on vasculature tone.^16^

Despite these prominent therapeutically exploitable advantages, clinical utilization of native PP as a positive NPY4R modulator is limited by a short circulatory half‐life of less than 7 min.^17^ This appears to be due to rapid cleavage of the N‐terminal dipeptide, Ala^1^‐Pro^2^, from PP by the ubiquitous enzyme dipeptidyl peptidase‐4 (DPP‐4).^18^ The DPP‐4 cleavage peptide, PP(3–36), is devoid of biological activity due largely to destabilization of the characteristic PP‐fold,^18^ a unique hairpin‐like structure essential for bioactivity of all NPY peptides.^19^ As such, DPP‐4 resistant PP molecules, that retain the established biological action profile of NPY4R activation, are required to fully uncover the promise of PP for obesity‐diabetes therapy.

Therefore, the present study examines the metabolic stability and biological activity of a series of PP analogs, that include amino acid substitution of Leu^3^ for Pro^3^ in PP, as well as N‐terminal and mid‐chain acylation, in an effort to curb DPP‐4 degradation and prolong half‐life. The enzymatic stability of these peptides, effects on beta‐cell function, growth, and survival as well as receptor dependency, was investigated in BRIN‐BD11 cells and compared to those of the native peptide. Furthermore, the impact of PP analogs on glucose tolerance, insulin secretion, and satiety was explored in normal mice, both alone and in combination with activation of glucagon‐like peptide‐1 (GLP‐1) or cholecystokinin (CCK) receptor signaling. The data demonstrate, for the first time, positive beta‐cell survival effects of enzymatically stabilized PP peptide analogs, coupled with sustained actions to reduce food intake, endorsing potential therapeutic promise of PP analogs for obesity‐derived forms of diabetes.

## 2 EXPERIMENTAL PROCEDURES

### 2.1 Peptides

All peptides were supplied by Synpeptide (Shanghai, China) at 95% purity. Purity and molecular mass were validated in‐house using reversed‐phased high‐performance liquid chromatography (rp‐HPLC) and matrix‐assisted laser desorption ionization time‐of‐flight mass spectrometry (MALDI‐ToF MS), respectively, as described.^11^ PP analogs synthesized included [P^3^]PP, [K^13^Pal]PP, [P^3^,K^13^Pal]PP, [N‐Pal]PP, and [N‐Pal,P^3^]PP, where Pal is palmitic acid (Table 1). A γ‐glutamyl spacer was employed to conjugate the C‐16 palmitate fatty acid (Pal) to the free amino group of K^13^ in [K^13^Pal]PP, and [P^3^,K^13^Pal]PP, or the free N‐terminal amino group in [N‐Pal]PP and [N‐Pal,P^3^]PP, as described previously.^20^, ^21^

**TABLE 1:** Amino acid sequence, molecular mass, purity, and in vitro DPP‐4 degradation profile of native PP and related analogs.

Peptide	Amino acid sequence	Expected mass (Da)	Calculated mass (Da)	Peptide purity	% intact peptide after 8 h DPP‐4 incubation
PP	APLEPVYPGDNATPEQMAQYAADLRRYINMLTRPRY‐NH_2_	4181.7	4182.7	>95	55.7
[P^3^]PP	AP**P**EPVYPGDNATPEQMAQYAADLRRYINMLTRPRY‐NH_2_	4165.7	4166.6	>95	>99
[K^13^Pal]PP	APLEPVYPGDNA**(K‐Pal)**PEQMAQYAADLRRYINMLTRPRY‐NH_2_	4566.2	4558.5	>95	>99
[P^3^,K^13^Pal]PP	AP**P**EPVYPGDNA**(K‐Pal)**PEQMAQYAADLRRYINMLTRPRY‐NH_2_	4550.2	4542.5	>95	>99
[N‐Pal]PP	(**N‐Pal**)APLEPVYPGDNATPEQMAQYAADLRRYINMLTRPR‐NH_2_	4437.1	4391.2	>95	>99
[N‐Pal,P^3^]PP	(**N‐Pal**)AP**P**EPVYPGDNATPEQMAQYAADLRRYINMLTRPR‐NH_2_	4421.1	4418.4	>95	>99

*Note*: The amino acid sequence of each peptide is listed using standardized single‐letter amino acid abbreviations. A γ‐glutamyl spacer was employed to conjugate the C‐16 palmitate fatty acid (Pal) to the free amino group of the peptide N‐terminus or K^13^, as appropriate. Changes from the native sequence are shown in bold text. Molecular mass was determined by MALDI‐MS analysis and peptide purity by rp‐HPLC. For DPP‐4 degradation studies, peptides (50 μg) were incubated with purified DPP‐4 enzyme (5 μL, 0.01 U/L) for 0–8 h. Reactions were terminated by the addition of trifluoroacetic acid (10% v/v) at either 0 or 8 h. Degradation profiles were then analyzed using rp‐HPLC with 214 nm UV absorbance and HPLC peaks of interest collected for subsequent MALDI‐MS analysis.

### 2.2 DPP‐4 degradation

PP peptides (50 μg) were incubated at 37°C for 0–8 h with 5 μL of purified human DPP‐4 enzyme (0.01 U/L, Sigma‐Aldrich, Gillingham, UK). Degradation reactions were terminated via the addition of 10 μL 10% (v/v) trifluoroacetic acid/water solution. Reaction mixes were separated by reverse phase rp‐HPLC using a Phenomenex C‐18 analytical column (250 × 4.6 mm^2^) with UV absorbance detection at 214 nm (ThermoQuest SpectraSystem UV2000 detector). HPLC peaks of interest were collected and analyzed by MALDI‐TOF MS on a Perseptive Biosystems Voyager‐DE Biospectrometry (Hertfordshire, UK).

### 2.3 In vitro insulin secretion

Effects of the PP and related peptides on insulin secretion were examined in pancreatic BRIN‐BD11 beta‐cells. The secretory characteristics of this cell line have been described in detail previously.^22^ Cells were cultured in RPMI 1640 media (Gibco Life Technologies Ltd), with a glucose concentration of 11.1 mM and supplemented with 10% v/v fetal bovine serum (Gibco), 1% v/v antibiotics (0.1 mg/mL streptomycin and 100 U/mL penicillin) at 37°C in 5% atmospheric CO_2_. For experimentation, cells were seeded into 24‐well plates (Falcon Ltd) at a density of 150,000 cells per well. Following overnight attachment, media was aspirated, and cells were preincubated in 1.1 mM glucose KRB buffer for 40 min. Following preincubation, the 1.1 mM glucose solution was removed and 1 mL of KRB test solution, containing 16.7 mM glucose with PP peptides (10^−12^ to 10^−6^ M) was added for 20 min. In a separate series, PP peptides were also incubated in the presence of alanine (10 mM) at 16.7 mM glucose to further investigate the effects on insulin secretion. For all experiments, following a 20 min incubation period, the supernatant was collected and stored at −20°C until insulin concentration determination using a dextran‐coated charcoal insulin radioimmunoassay.^23^

### 2.4 Beta‐cell proliferation and apoptosis

To assess the effects of PP and related peptides (10^−8^ and 10^−6^ M) on beta‐cell proliferation and apoptosis, BRIN‐BD11 beta‐cells were seeded onto sterilized, clear‐glass coverslips (16 mm diameter) and placed in 12‐well plates (Falcon Ltd) at a density of 40,000 cells per well and cultured for 18 h. Media control (11.1 mM glucose), GLP‐1 (10^−8^ and 10^−6^ M), and a cytokine cocktail mix (IL‐1β [100 U/mL], IFNγ [20 U/mL], TNFα [200 U/mL], Sigma Aldrich, UK) were employed as controls, as appropriate. Cells were then rinsed with PBS and fixed using 4% paraformaldehyde. After antigen retrieval with sodium citrate buffer at 95°C for 20 min, blocking was performed using 2% bovine serum albumin (BSA) for 45 min. For proliferation studies, the slips were then incubated at 37°C with rabbit anti‐Ki‐67 primary antibody (Abcam, ab15580), and subsequently with Alexa Fluor® 488 secondary antibody. Coverslips were mounted onto polylysine‐coated microscope slides using a 50:50 glycerol:PBS solution and stored at 4°C until required for analysis. To assess the ability of PP peptides to protect against cytokine‐induced apoptosis, cells were seeded, washed, and fixed as above, with the exception that the media was supplemented with the cytokine mix. The slips were then incubated at 37°C with TUNEL reaction mix for 60 min (Roche Diagnostics, Germany), and mounted onto microscope slides. To assess receptor specificity, co‐incubation of PP peptides with the well characterized NPY4R antagonist, (S)‐VU0637120,^24^ at 10^−5^ M was employed for beta‐cell proliferation experiments, conducted as detailed above. All slides were viewed using a fluorescent microscope (Olympus System Microscope, model BX51; Southend‐on‐Sea, UK) and photographed by DP70 camera adapter system. Proliferation/TUNEL positive frequency was determined using the cell‐counter function on ImageJ Software and expressed as % of total cells analyzed.

### 2.5 In vivo experiments

Acute in vivo studies were conducted in groups (*n* = 6) of 4‐month‐old male C57BL/6 mice (Envigo, Huntington, UK). All mice were maintained on standard rodent chow (10% fat, 30% protein, and 60% carbohydrate; Trouw Nutrition, Northwich, UK), and drinking water provided ad libitum. Mice were housed individually and kept in a temperature‐controlled environment (22 ± 2°C), with a 12‐h light/dark cycle. Experiments were carried out in accordance with the UK Animal Scientific Procedures Act 1986 as well as being approved by Ulster University Animal Ethics Review Committee (AWERB) committee and protected by the UK Home Office Animal project license number PPL2902, approved on April 26, 2021. Acute effects of test peptides on food intake were tested in overnight (16 h) fasted mice. Briefly, fasted mice were administered an intraperitoneal (i.p.) injection of saline vehicle (0.9% (w/v) NaCl) alone or in combination with test peptides (each at 25, 75, or 200 nmol/kg bw) and cumulative food intake measured at regular intervals, to assess the pharmacodynamic profile of the test peptides. For food intake studies, mice were provided with preweighed rodent maintenance diet in their food hopper at *t* = 0 min, which was then re‐weighed at each timepoint with all due care taken to account for any wastage or crumbling of the food pellets. In this regard, the investigator was present at all times during the 180‐min test period to ensure accuracy and consistency in all measurements. Furthermore, effects of PP or related peptides (25 nmol/kg bw) on glucose homeostasis and insulin secretion were evaluated in overnight fasted mice following i.p. injection of glucose alone (18 mmol/kg bw) or in combination with PP peptides (each at 25 nmol/kg bw). In a separate series, the effects of combined administration of PP or related peptides (each at 25 nmol/kg bw) together with either exendin‐4 (0.25 nmol/kg bw) or CCK‐8 (25 nmol/kg bw) on food intake and glucose tolerance were also examined in mice, as described above. The doses of exendin‐4 and CCK‐8 were chosen based on previous studies reporting good efficacy of these peptides at the respective doses.^25^, ^26^

### 2.6 Biochemical analyses

Blood samples were collected from the cut tip on the tail vein, of conscious mice, at times indicated in figures. Blood glucose was measured immediately using a hand‐held Ascencia Contour blood glucose meter (Bayer Healthcare, Newbury, Berkshire, UK). For plasma insulin, blood was collected in chilled fluoride/heparin‐coated microcentrifuge tubes (Sarstedt, Numbrecht, Germany) and centrifuged using a Beckman microcentrifuge (Beckman Instruments, Galway, Ireland) for 10 min at 12,000 rpm. Plasma was separated and stored at −20°C, until determination of plasma insulin by radioimmunoassay.^23^

### 2.7 Statistical analysis

Statistical analyses were performed using GraphPad PRISM software (version 5.0). Values are expressed as mean ± S.E.M. Comparative analyses between groups were carried out using a one‐way analysis of variance (ANOVA) with Bonferroni's post hoc test. The difference between groups was considered significant if *p* < 0.05.

## 3 RESULTS

### 3.1 In vitro DPP‐4 stability

As expected, incubation of native PP with DPP‐4 for 8 h resulted in N‐terminal di‐peptide cleavage to yield PP(3–36) (Table 1). In contrast, all PP analogs were resistant to DPP‐4 mediated degradation for up to 8 h (Table 1).

### 3.2 Effects of PP and related peptides on in vitro insulin secretion

**FIGURE 1:**
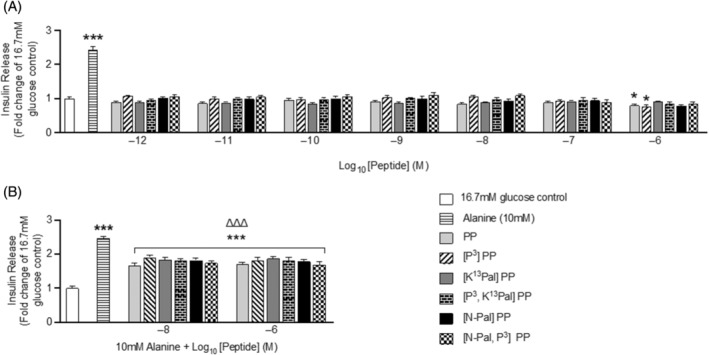
Effects of PP and related analogs on insulin secretion from BRIN‐BD11 beta‐cells. BRIN‐BD11 cells were incubated (20 min) with (A) 16.7 mM glucose or (B) 16.7 mM glucose supplemented with alanine (10 mM) and the effects of PP peptides (10^−12^ to 10^−6^ M) on insulin secretion determined. Values are mean ± SEM (*n* = 8). *p < 0.05, ***p < 0.001 compared with 16.7 mM glucose control. ^ΔΔΔ^ *p* < 0.001 compared with 16.7 mM glucose plus 10 mM alanine.

Native PP and related analogs exhibited no discernible impact on insulin secretion from BRIN‐BD11 cells at 16.7 mM glucose over the concentration range of 10^−12^ to 10^−7^ M (Figure 1A). A slight inhibitory effect of native PP and [P^3^]PP was observed at 10^−6^ M (Figure 1A). As shown in Figure 1B, all peptides significantly (*p* < 0.001) inhibited 10 mM alanine‐induced insulin secretion (Figure 1B).

### 3.3 Effects of PP and related peptides on BRIN‐BD11 cell proliferation and protection against apoptosis

**FIGURE 2:**
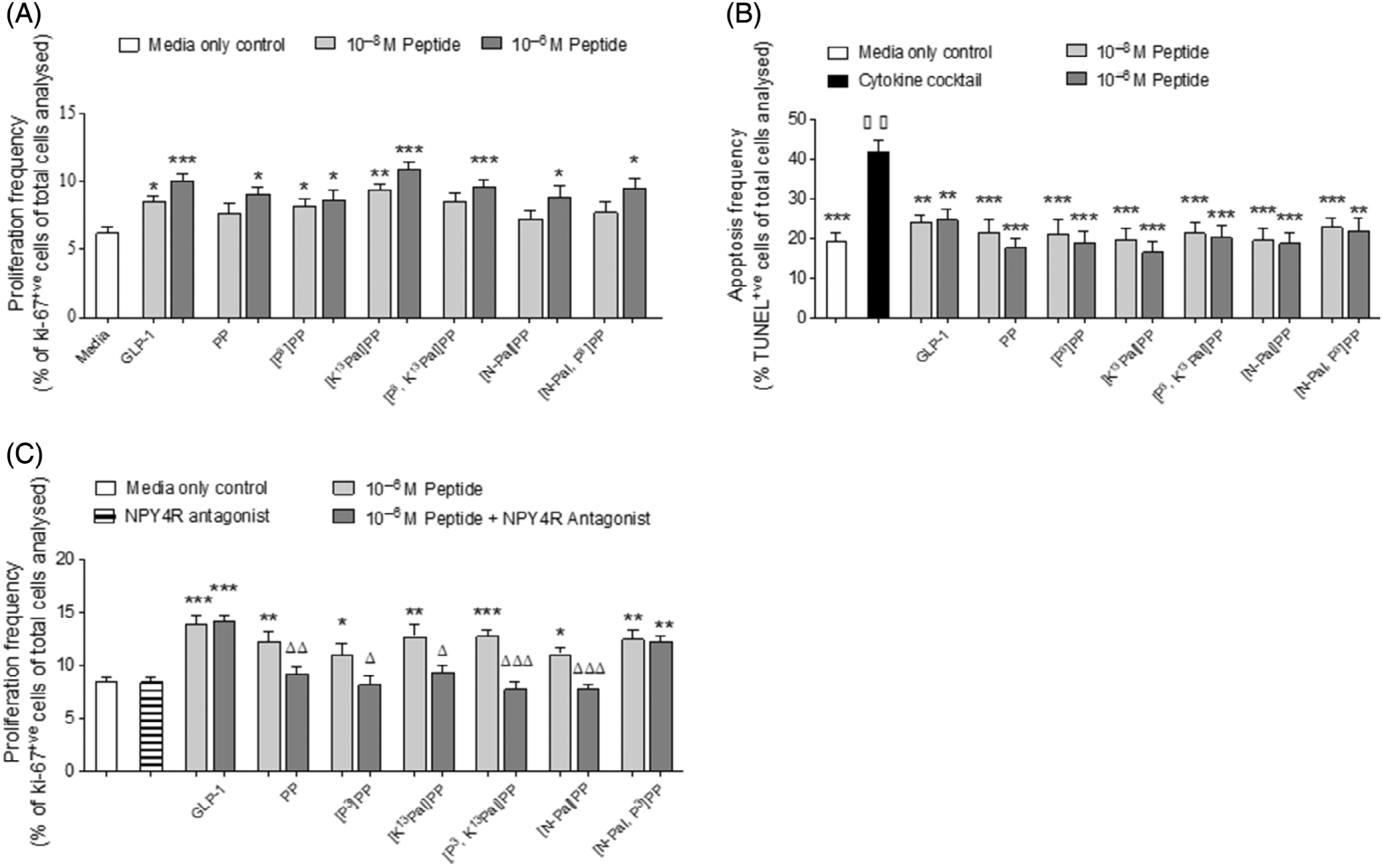
Effects of PP and related analogs on BRIN‐BD11 beta‐cell proliferation and protection from cytokine‐induced apoptosis. (A) BRIN‐BD11 cells were cultured (16 h) with PP peptides or GLP‐1 (10^−8^ to 10^−6^ M) and proliferation assessed by Ki‐67 staining. (B) BRIN‐BD11 cells were cultured (16 h) with PP peptides or GLP‐1 (10^−8^ to 10^−6^ M) in the presence of a cytokine cocktail (IL‐1β (100 U/mL), IFNγ (20 U/mL), TNFα (200 U/mL)) and apoptosis detected using the TUNEL assay. (C) BRIN‐BD11 cells were cultured (16 h) with PP peptides alone (10^−6^ M), or in the presence of the NPY4R antagonist (S)‐VU0637120 and proliferation assessed by Ki‐67 staining. All values are mean ± SEM (*n* = 3). (A–C) *p < 0.05, **p < 0.01, ***p < 0.001 compared with respective control. (C) ^Δ^ *p* < 0.05, ^ΔΔ^ *p* < 0.01, ^ΔΔΔ^ *p* < 0.001 compared with incubation with (S)‐VU0637120.

Native PP (10^−6^ M), similar to GLP‐1 positive control at 10^−8^ and 10^−6^ M, significantly (*p* < 0.001) enhanced BRIN‐BD11 cell proliferation when compared with control cultures (Figure 2A). Each of the PP analogs also enhanced (*p =* <0.05 to 0.001) beta cell proliferation at 10^−6^ M (Figure 2A). Moreover, [P^3^]PP and [K^13^Pal]PP augmented (*p* < 0.05 and *p* < 0.01, respectively) BRIN‐BD11 beta‐cell growth at 10^−8^ M (Figure 2A). In terms of protection against cytokine‐induced beta‐cell apoptosis, GLP‐1 and all PP peptides at both 10^−8^ and 10^−6^ M, significantly (*p =* <0.01 to 0.001) protected against cell death with similar efficacy (Figure 2B). Notably, beneficial beta‐cell proliferative benefits of native PP and all related analogs, with the exception of [N‐Pal,P^3^]PP, were shown to be dependent on NPY4R signaling following co‐incubation with the specific NPY4R antagonist (S)‐VU0637120 (Figure 2C).

### 3.4 Effects of PP and related peptides on food intake in mice

**FIGURE 3:**
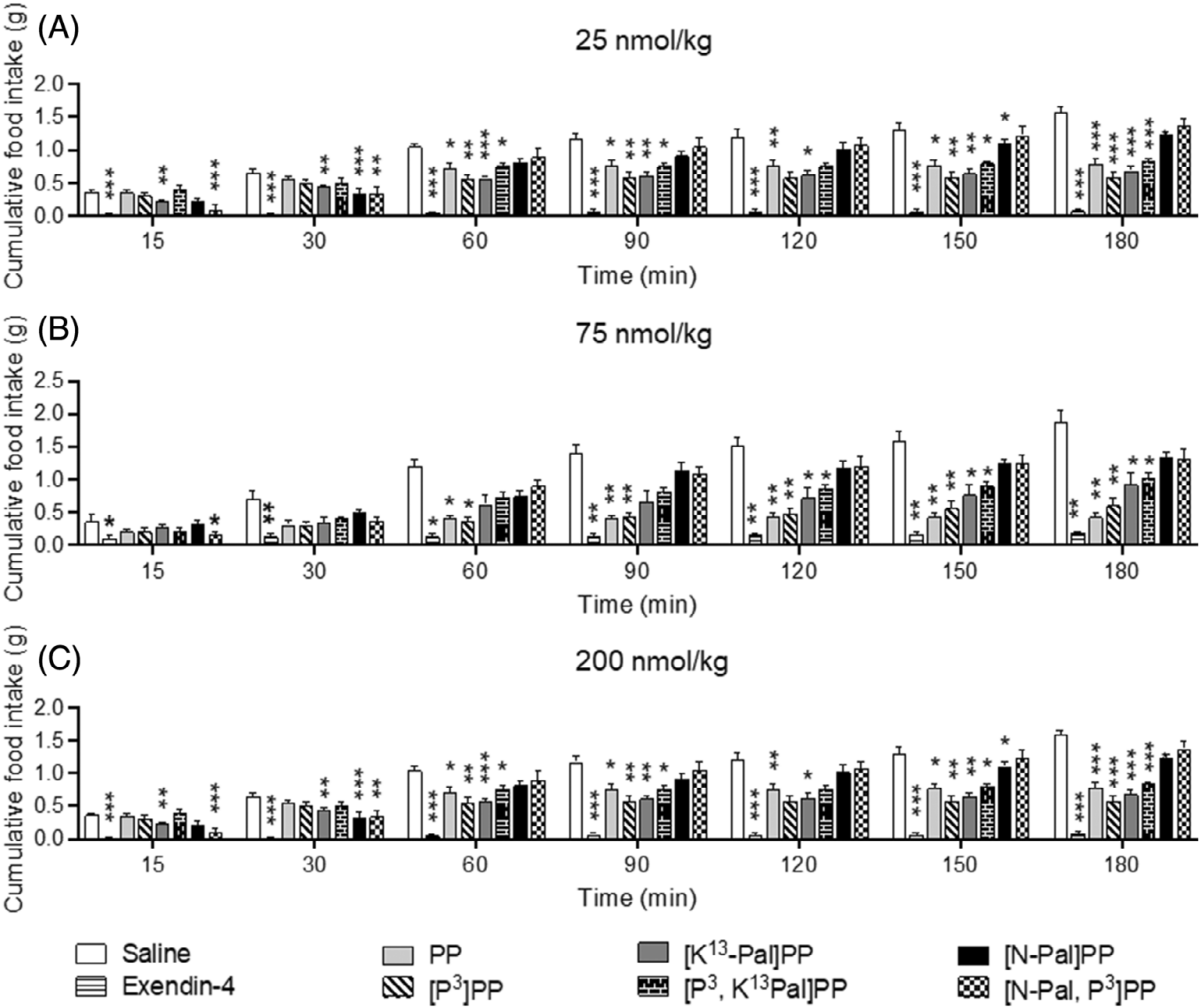
Dose‐dependent effects of PP and related analogs on food intake in mice. Cumulative food intake was assessed after i.p. administration of saline vehicle (0.9% NaCl) alone or in combination with PP peptides at (A) 25, (B) 75, and (C) 200 nmol/kg body weight. All values are mean ± SEM (*n* = 6). *p < 0.05, **p < 0.01, ***p < 0.001 compared with respective saline control.

**FIGURE 4:**
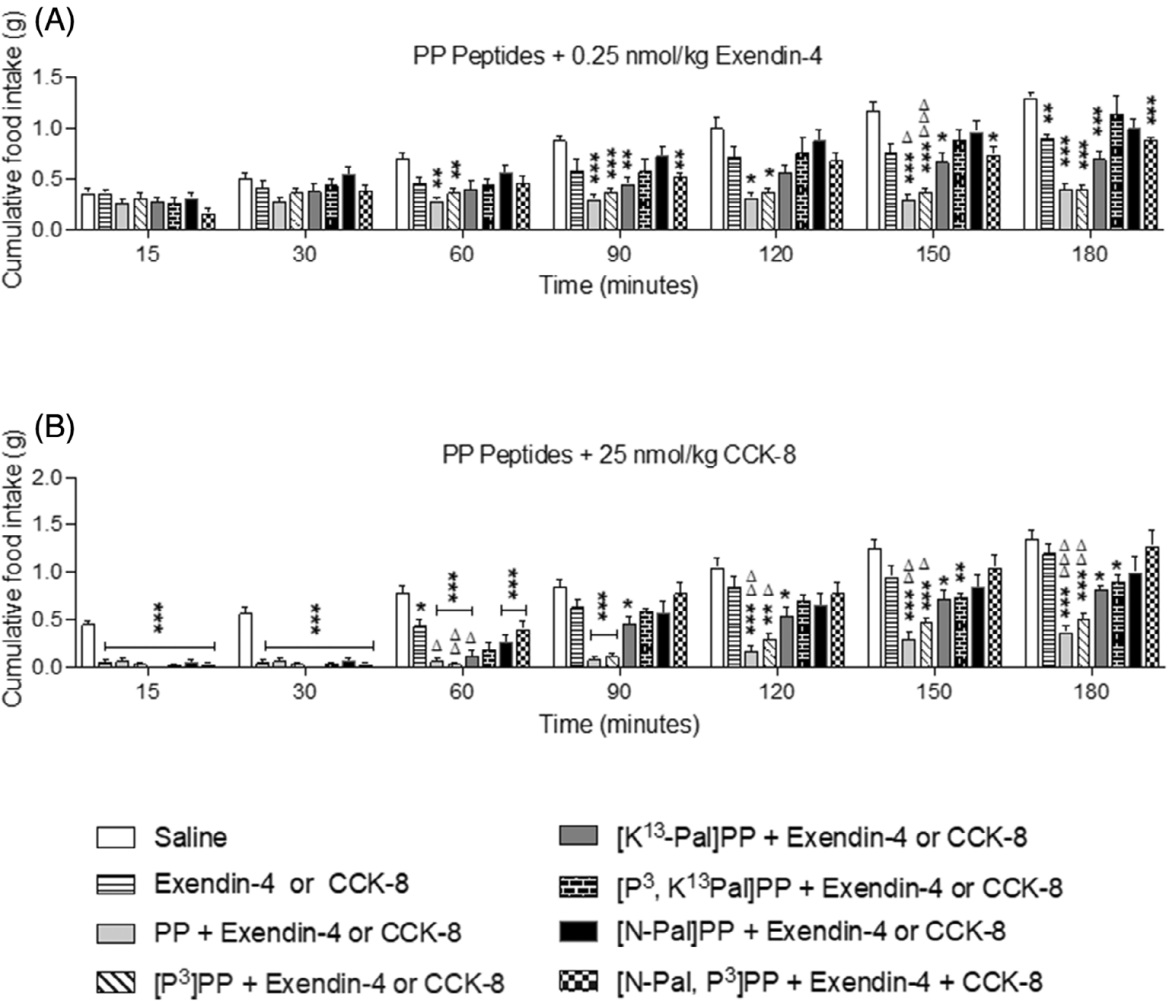
Effects of PP and related analogs on food intake in combination with exendin‐4 or CCK‐8 in mice. Cumulative food intake was assessed after i.p. administration of saline vehicle (0.9% NaCl) or in combination with PP peptides (25 nmol/kg body weight) in combination with (A) exendin‐4 (0.25 nmol/kg body weight) or (B) CCK‐8 (25 nmol/kg body weight). All values are mean ± SEM (*n* = 6). *p < 0.05, **p < 0.01, ***p < 0.001 compared with saline control. ^∆^ *p* < 0.05, ^∆∆^ *p* < 0.01, ^∆∆∆^ *p* < 0.001 compared with (A) exendin‐4 or (B) CCK‐8.

At a dose of 25 nmol/kg, all test peptides, except [N‐Pal]PP and [N‐Pal,P^3^]PP, induced significant (*p =* <0.05 to <0.001) appetite suppressive actions at various study time points in mice previously fasted overnight (Figure 3A). Interestingly, native PP and [P^3^]PP were particularly effective in this regard (Figure 3A). Similar observations were made when the peptides were administered at a dose of 75 nmol/kg (Figure 3B). At 200 nmol/kg, [N‐Pal]PP did exert a mild inhibitory (*p* < 0.05) effect on food intake at the later observation points, but [N‐Pal,P^3^]PP lacked bioactivity within this experimental system at all doses employed (Figure 3A–C). In terms of additive appetite suppressive actions, this was particularly evident when native PP, [P^3^]PP or [K^13^Pal]PP were administered with 0.25 nmol/kg exendin‐4 (Figure 4B). Interestingly, CCK‐8 exerted impressive effects on appetite suppression (*p* < 0.001) at the early observation points, with native PP and [P^3^]PP able to significantly (*p =* <0.05 to 0.01) augment this effect from 90 min onwards (Figure 4C).

### 3.5 Effects of PP peptides on glucose tolerance and insulin secretion in mice

**FIGURE 5:**
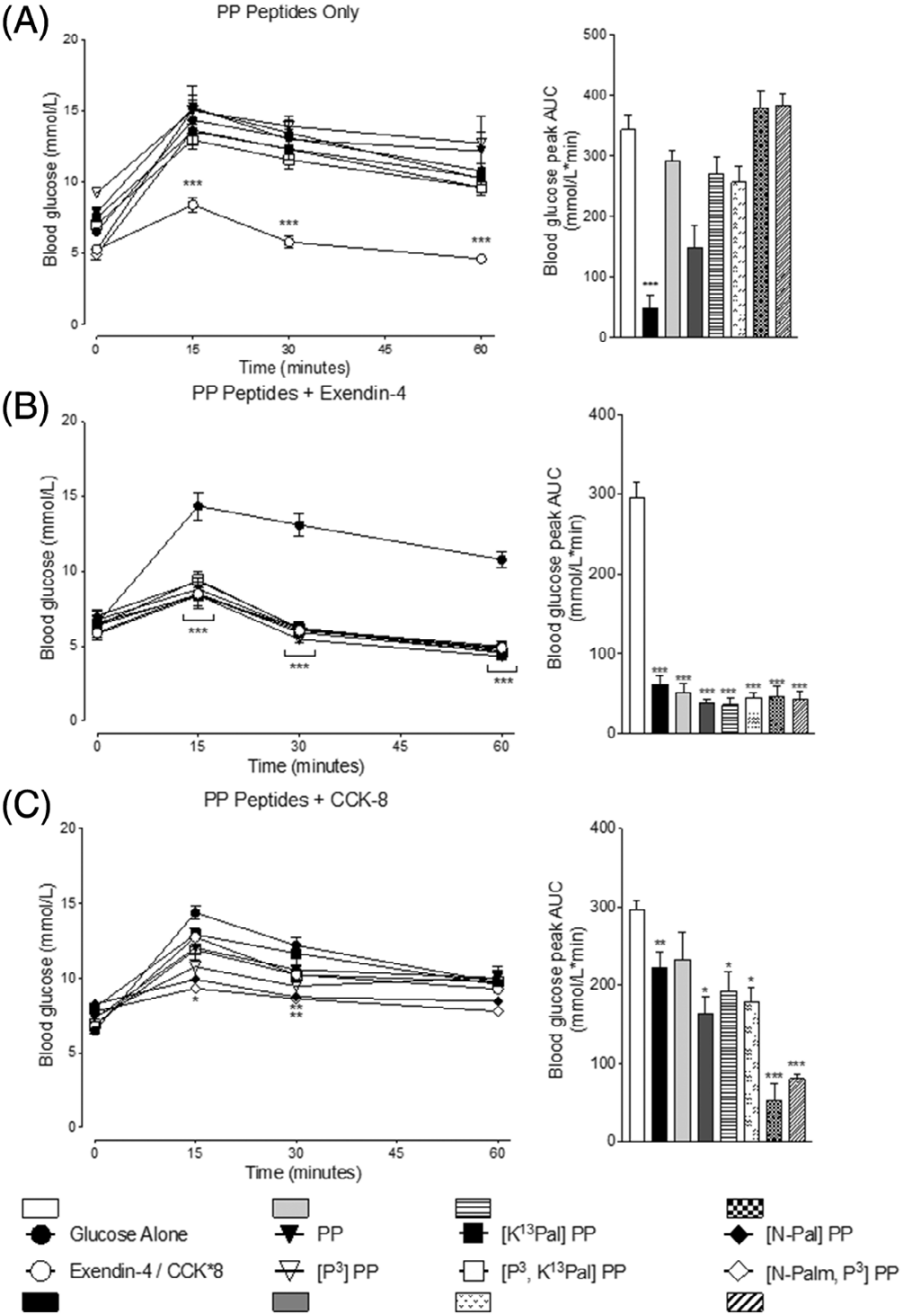
Effects of PP and related analogs on glucose tolerance alone and in combination with exendin‐4 or CCK‐8 in mice. (A–C) Blood glucose was assessed immediately prior to, and at regular intervals subsequent to, i.p. injection of PP peptides (each at 25 nmol/kg body weight dose) with glucose (18 mmol/kg body weight) either (A) alone or in combination with (B) exendin‐4 (0.25 nmol/kg body weight) or (C) CCK‐8 (25 nmol/kg body weight). All associated 0–60 min AUC data are presented with individual baseline subtraction employed. All values are mean ± SEM (*n* = 6). *p < 0.05, **p < 0.01, ***p < 0.001 compared with glucose control.

**FIGURE 6:**
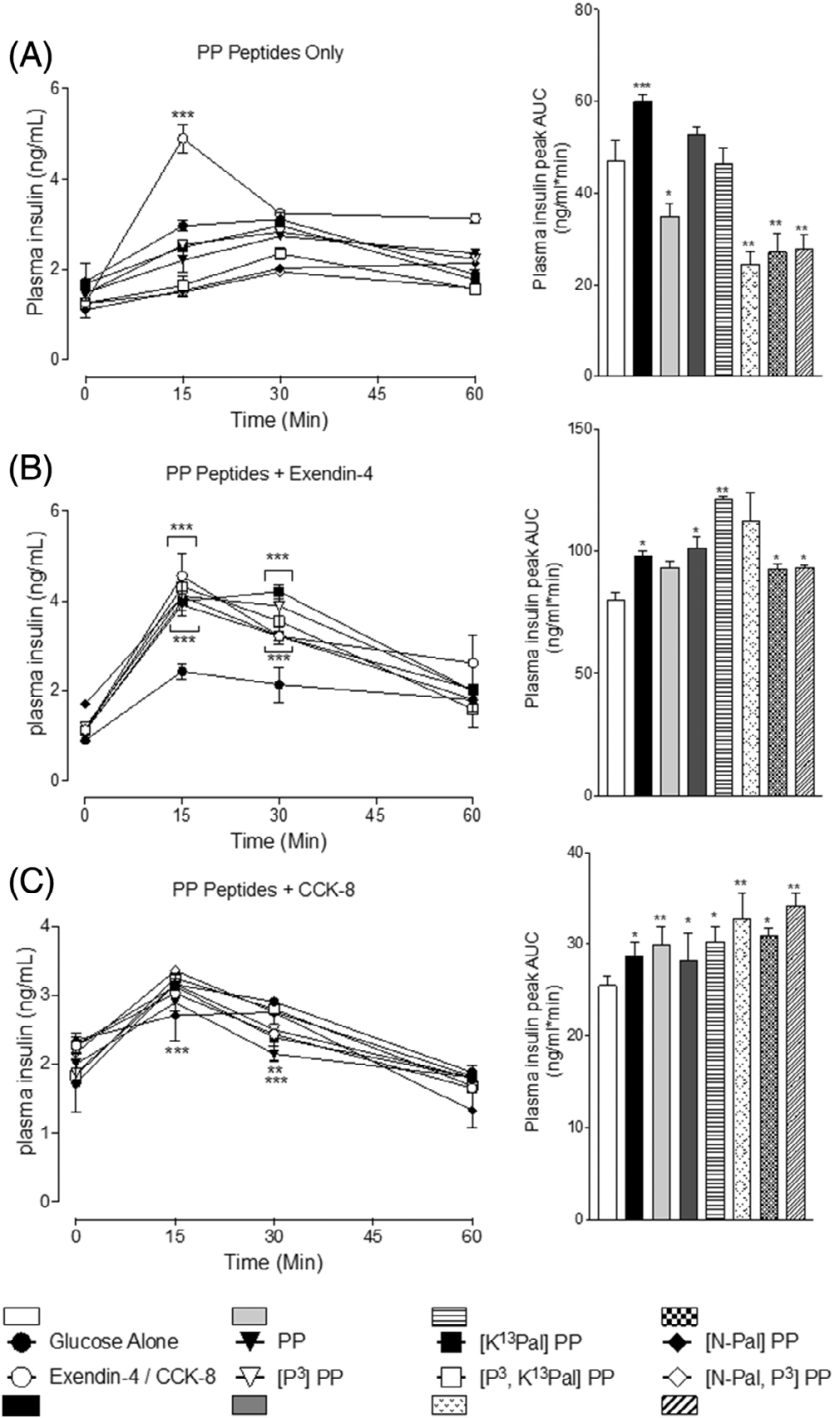
Effects of PP and related analogs on glucose‐stimulated insulin secretion alone and in combination with exendin‐4 or CCK‐8 in mice. (A–C) Plasma insulin was assessed immediately prior to, and at regular intervals subsequent to, i.p. injection of PP peptides (each at 25 nmol/kg body weight dose) with glucose (18 mmol/kg body weight) either (A) alone or in combination with (B) exendin‐4 (0.25 nmol/kg body weight) or (C) CCK‐8 (25 nmol/kg body weight). All associated 0–60 min AUC data are presented with individual baseline subtraction employed. All values are mean ± SEM (*n* = 6). *p < 0.05, **p < 0.01, ***p < 0.001 compared with glucose control.

Neither native PP, nor related PP analogs, exerted any effect on glucose homeostasis when administered conjointly with glucose to mice (Figure 5A). However, the PP peptides did not perturb the benefits of exendin‐4 or CCK‐8 (*p =* <0.05 to <0.001) on glucose disposal in mice (Figure 5B,C), with the exception of [P^3^,K^13^Pal]PP that appeared to mildly impair the overall glucose‐lowering action of CCK‐8 (Figure 5C). In terms of glucose‐induced insulin secretion, the PP analogs again exhibited limited impact with native PP, [P^3^,K^13^Pal]PP, [N‐Pal]PP, and [N‐Pal,P^3^]PP even slightly decreasing (*p* = <0.05 to 0.01) this parameter (Figure 6A). However, importantly the PP peptides did not interfere with the significant insulinotropic actions of exendin‐4 or CCK‐8 (Figure 6B,C).

## 4 DISCUSSION

Metabolic advantages of sustained activation of the NPY1R and NPY2R have previously been documented,^22^, ^27^ whereas related benefits of NPY4R activation are less well understood.^28^ This is despite the knowledge that the NPY4R can induce direct pancreatic beta‐cell survival benefits^10^ as well as suppress appetite.^29^ However, the endogenous NPY4R ligand, namely PP, has an extremely short circulating half‐life due to rapid degradation by the ubiquitous hormone DPP‐4,^18^ which precludes therapeutic utility. Thus, enzyme‐resistant forms of PP are required to fully uncover potential metabolic benefits, an approach which has also been successfully applied to related DPP‐4 susceptible peptide hormones such as glucose‐dependent insulinotropic polypeptide (GIP), GLP‐1, and peptide tyrosine tyrosine (PYY).^30^, ^31^, ^32^

In this respect, we adopted well‐established approaches to generate longer acting PP analogs that included amino acid substitution and/or acylation. As such, incorporation of a proline residue at position 3 of DPP‐4 liable peptides protects against enzymatic degradation,^33^ while acylation also extends pharmacodynamic profile by promoting albumin binding.^34^ In good agreement, lipidation of native PP using various fatty acid hydrocarbon chain lengths, has been reported to extend pharmacokinetic profile without affecting receptor selectivity.^35^ Our studies with [K^13^Pal]PP, [P^3^,K^13^Pal]PP, [N‐Pal]PP, and [N‐Pal,P^3^]PP confirm these initial observations, generating acylated PP analogs that, to the most part, maintain Y4 selectivity at the level of the pancreatic beta‐cell. However, our utilization of a γ‐glutamyl spacer molecule between the peptide chain and fatty acid moiety, which is considered important for distancing the active peptide from albumin and maintaining peptide potency,^36^ was not employed in the early studies by Mäde et al.,^37^ perhaps reflecting why bioactivity of those compounds remained undetermined. In addition to this, a longer‐acting PEGylated form of PP has also been documented and shown to decrease appetite in mice.^37^ In this regard, acylation of regulatory peptide hormones has proven to be more successful than PEGylation,^38^, ^39^, ^40^ likely related to decreased target potency as the PEG mass increases,^41^ that is not apparent with fatty acid derivation.^36^ Moreover, the impact of all previously described PP analogs on pancreatic beta‐cell function has not been explored.

Considering activation of beta‐cell NPY1R induces notable benefits on beta‐cell growth and survival,^42^ coupled with the knowledge that NPY1R and NPY4R stimulate comparable cell signaling pathways,^43^, ^44^ it was not unexpected that all novel PP analogs augmented beta‐cell survival and protected against cytokine‐induced apoptosis in the current study. We confirmed that such benefits were directly related to specific NPY4R activation for all PP analogs, barring [N‐Pal,P^3^]PP, through the use of (S)‐VU0637120 as a well characterized NPY4R antagonist,^24^ although peptide NPY4R binding affinity still remains to be determined. Although PP circulates at picomolar concentrations,^17^ it is likely that local islet concentrations are much higher. To account for this, and the lack of paracrine interactions within the in vitro environment, we employed micromolar concentrations of PP and related analogs for studies in BRIN‐BD11 cells. While modulation of pancreatic islet NPY1R and NPY4R leads to insulinostatic effects,^10^, ^12^ that was affirmed by all novel PP analogs, induction of beta‐cell rest can be viewed as advantageous under situations of heightened metabolic demand and beta‐cell stress, helping to protect beta‐cell health and improve enduring glycaemic control.^45^, ^46^ This becomes particularly significant since beta‐cells are recognized to have relatively poor antioxidant defense capabilities.^47^ It is interesting that insulinostatic effects of the PP peptides were more apparent in the presence of alanine rather than glucose, both of which would activate similar secretion coupling pathways in beta‐cells.^22^ Consequently, this difference likely relates to the enhanced capability of alanine to elevate intracellular Ca^2+^ due to co‐transport with Na^+^ resulting in particularly powerful stimulation of insulin secretion from BRIN‐BD11 cells.^48^ In addition to this, NPY1R activation has recently been demonstrated to drive pancreatic alpha‐ to beta‐cell transdifferentiation in severely diabetic mice, in a bid to restore beta‐cell mass.^12^ In good harmony with this, PP‐positive cells within the islet are recognized as potential progenitors for beta‐cell restoration,^49^, ^50^ suggesting likely comparable benefits of PP‐derived peptides on islet cell lineage transition. Taken together, the predicted value in targeting pancreatic NPY4R over NPY1R is also related to a lack of off‐target effects to increase vasculature tone, which has been observed with prolonged NPY1R activation.^51^ Moreover, whereas NPY1R activation can lead to stimulation of appetite,^51^ NPY4R has well characterized anorectic actions.^52^

In this context, [P^3^] PP, [K^13^Pal]PP, and [P^3^,K^13^Pal]PP retained impressive effects to restrict food intake in overnight fasted mice. The N‐terminally acylated peptides, namely [N‐Pal]PP and [N‐Pal,P^3^]PP, were devoid of similar bioactivity. Since NPY4R‐dependent activity of these peptides was observed in vitro, alongside their enhanced enzymatic stability, the most likely explanation for this difference would appear to be a passage through the blood–brain barrier or peptide aggregation at the injection site. However, further detailed study would be required to confirm this, that are outside the scope of the current study. The PP peptide doses employed for in vivo studies were selected based on previous positive experience with enzyme‐resistant regulatory endocrine peptides.^11^, ^12^, ^20^, ^25^, ^30^, ^31^, ^32^, ^33^, ^34^

Given a recent upsurge of interest in the therapeutic applicability of dual‐ or triple‐acting hybrid peptides,^53^ we also investigated satiety effects of PP analogs in combination with either GLP‐1 or CCK, as well recognized anorectic agents.^54^ This was also encouraged by our related investigations that confirmed the PP peptides did not perturb their glucose‐stimulated insulin‐releasing actions in vivo. While the appetite suppressive benefits of GLP‐1 receptor activation in combination with various other related regulatory hormones such as, but not limited to, CCK,^53^ PYY(3–36),^55^ and GIP^56^ have been described in detail, similar benefits with PP are less well characterized. In this study, native PP as well as [P^3^]PP exerted notable additive satiety benefits when co‐administered with exendin‐4 to overnight fasted mice. Similar effects of these PP peptides were also noted in combination with CCK, and it is noteworthy that this hormone stimulates PP secretion.^28^ Thus, there appear to be therapeutically exploitable interactions between PP and such hormones that merit further investigation. Importantly, PP and the analogs tested did not affect glucose tolerance or plasma insulin responses when given alone or in combination with exendin‐4 or CCK‐8.

### 4.1 Conclusions

The present study demonstrates that long‐acting, enzymatically stable, PP peptides with NPY4R specificity can be generated. Notably, all PP analogs retained the advantageous insulinostatic and beta‐cell resting benefits of native PP,^10^ linked to longer term protection of beta‐cell health.^45^, ^46^ Interestingly, N‐terminal palmitoylation appeared to disturb in vivo effectiveness, but substitution of Leu^3^ for Pro^3^, and/or mid‐chain acylation, created PP analogs with potential prominent benefits on pancreatic islet architecture and energy regulation that merit increased scrutiny for the treatment of diabetes and obesity. In addition, the exploration of combination therapies involving PP and other regulatory peptide hormones constitutes another compelling avenue for further investigation.

## AUTHOR CONTRIBUTIONS

Peter R. Flatt and Nigel Irwin conceived/designed the study. Ryan A. Lafferty, Nigel Irwin, and Wuyun Zhu drafted the manuscript. Ryan A. Lafferty, Neil Tanday, and Wuyun Zhu participated in the conduct/data collection and analysis and interpretation of data. All authors revised the manuscript critically for intellectual content and approved the final version of the manuscript.

## CONFLICT OF INTEREST STATEMENT

Peter R. Flatt and Nigel Irwin are named on patents for the exploitation of peptide‐based drugs for diabetes. Ryan A. Lafferty, Peter R. Flatt, and Nigel Irwin are shareholders in Dia Beta Labs Ltd. Wuyun Zhu and Neil Tanday declare no conflict of interest.

## Data Availability

The authors declare that the data supporting the findings of this study are available within the article. Any additional raw data supporting the conclusions of this article will be made available by the senior author (Nigel Irwin), without undue reservation.
